# The UVB-Stimulated Expression of Transglutaminase 1 Is Mediated Predominantly via the NFκB Signaling Pathway: New Evidence of Its Significant Attenuation through the Specific Interruption of the p38/MSK1/NFκBp65 Ser276 Axis

**DOI:** 10.1371/journal.pone.0136311

**Published:** 2015-08-25

**Authors:** Shuko Terazawa, Shingo Mori, Hiroaki Nakajima, Michitaka Yasuda, Genji Imokawa

**Affiliations:** 1 Research Institute for Biological Functions, Chubu University, Aichi, Japan; 2 School of Bioscience and Biotechnology, Tokyo University of Technology, Tokyo, Japan; Faculty of Medicine & Health Sciences, UNITED ARAB EMIRATES

## Abstract

The influence of ultraviolet B (UVB) radiation on transglutaminase 1 (TGase 1), a major factor that regulates skin keratinization, has not been sufficiently characterized especially at the gene or protein level. Thus, we determined whether UVB affects the expression of TGase 1 in human keratinocytes and clarified the intracellular stress signaling mechanism(s) involved. Exposure of human keratinocytes to UVB significantly up-regulated the expression of TGase 1 at the gene and protein levels. Treatment with inhibitors of p38, MEK, JNK or NFκB significantly abolished the UVB-stimulated protein expression of TGase 1. Treatment with astaxanthin immediately after UVB irradiation did not attenuate the increased phosphorylation of Ser536/Ser468NFκBp65, c-Jun, ATK-2 and CK2, and did not abrogate the increased or diminished protein levels of c-Jun/c-Fos or I-κBα, respectively. However, the same treatment with astaxanthin significantly abolished the UVB-stimulated expression of TGase 1 protein, which was accompanied by the attenuated phosphorylation of Thr565/Ser376/Ser360MSK1, Ser276NFκBp65 and Ser133CREB. The MSK1 inhibitor H89 significantly down-regulated the increased protein expression of TGase 1 in UVB-exposed human keratinocytes, which was accompanied by an abrogating effect on the increased phosphorylation of Ser276NFκBp65 and Ser133CREB but not Thr565/Ser376/Ser360MSK1. Transfection of human keratinocytes with MSK1 siRNA suppressed the UVB-stimulated protein expression of TGase 1. These findings suggest that the UVB-stimulated expression of TGase 1 is mediated predominantly via the NFκB pathway and can be attenuated through a specific interruption of the p38/MSK1/NFκBp65Ser276 axis.

## Introduction

Exposure of the skin to ultraviolet B (UVB) radiation causes inflammation and subsequent hyperkeratosis of the epidermis [1]. Hyperkeratotic skin is characterized by a roughened and toughened surface due to the formation of a hardened and thickened cornified cell envelope. Intercellular lipids between layers of the stratum corneum, especially ceramides that play an important role in retaining water by forming lamellar structures, serve as a lubricant for the stratum corneum layers. The ceramide level in the stratum corneum is known to be markedly up-regulated within several days after UVB radiation [2]. Since the UVB-induced roughened skin could not be reasonably accounted for by the increased level of ceramides in the stratum corneum, little is known about the mechanism(s) involved in UVB-induced effects that result in the roughened and toughened skin. We hypothesized that the UVB-induced roughened skin might result from a thickened cornified cell envelope, which could be caused by an increase in the enzyme activity of transglutaminase(s) (TGases).

TGases are Ca^2+-^dependent enzymes which catalyze ε-(γ-glutamyl)lysine cross-linking reactions. Four TGases (1, 2, 3 and X) are constitutively expressed in epithelial tissues such as the epidermis [3, 4], and TGase 1 and TGase 3 have been shown to play essential roles in epidermal keratinization [5, 6, 7]. TGase 1 predominantly exists in the upper spinous and granular layers of the skin [8, 9] and serves as a membrane-bound TGase isozyme [10], whose role is associated mainly with generation of the cross-linked cell envelope in epidermal keratinocytes [11, 12]. Mutations of the gene encoding membrane-bound TGase 1 elicit an autosomal recessive skin disorder known as lamellar ichthyosis, which results from an aberrant stratum corneum with the lipid and cornified envelopes being seriously injured [13, 14]. In mice lacking the gene encoding TGase 1, lipid lamellar granules and cornified envelopes are not generated, leading to a severely disrupted skin barrier [15]. TGase 1 can also catalyze the formation of ester bonds between specific glutaminyl residues of human involucrin and epidermal specific omega-hydroxyceramides [16], which also play an important role in normal skin barrier function. On the other hand, TGase 3 is a soluble enzyme expressed predominantly in differentiating keratinocytes, corneocytes and hair follicles [17]. A recent study of TGase 3 knockout mice demonstrated that they have no distinct abnormality in skin development, no unequivocal aberration in barrier function or in the potential to heal wounds [18]. On the other hand, hairs produced in mice lacking TGase 3 are thinner, showing marked alterations in the cuticle cells with hair protein cross-linking being distinctly attenuated. Therefore, it is likely that TGase 3 is required for proper hair development, but not for formation of the cornified cell envelope and the epidermal barrier.

As for studies examining the effect of UVB on TGase 1 in the epidermis, Takahashi et al. [19] reported that UVB does not induce membrane TGase activity in cultured primary human keratinocytes. On the other hand, Del Bino et al. [1] have clearly shown that UVB induces hyperplasia of the epidermis with an over-expressed immuno-stainable TGase 1. Since the activation of TGase 1 is required for its subsequent proteolytic processing by cathepsin D or other proteinases [20], those earlier studies characterizing the enzymatic activity or immunostaining of TGase 1 have limitations for elucidating the effect of UVB on TGase 1 in vivo. Thus, to understand the differentiation process of human keratinocytes after UVB exposure, it is important to determine whether UVB can stimulate the expression of TGase 1 in human keratinocytes at the gene and/or protein level and to elucidate the intracellular signaling mechanism(s) by which TGase 1 expression is regulated by UVB irradiation. In the present study, we characterized the signaling mechanisms underlying the UVB-increased expression of TGase 1 by evaluating the effects of several inhibitors of stress-activated signaling factors. We also utilized the differential actions of astaxanthin (AX) on signaling pathways downstream of those stress-activated signaling pathways when treated before or after UVB exposure [21]. Our results show that UVB stimulates TGase 1 expression predominantly via the activation of NFκB in human keratinocytes, which can be attenuated even by post-irradiation treatment with H89 or AX or by the silencing of mitogen- and stress-activated protein kinase (MSK1) through a specific interruption of p38/MSK1/NFκBp65 axis in a reactive oxygen species (ROS) depletion-independent manner.

## Materials and Methods

### Materials

Serum-free keratinocyte growth medium (Medium 154S) containing low calcium (0.2 mM), bovine pituitary extract (BPE) and epidermal growth factor (EGF) were obtained from Kurabo (Tokyo, Japan). Human primary keratinocytes (HPKs) were purchased from Biopredic Intl (Saint-Gregoire, France). Human HaCaT keratinocytes [22, 23] were kindly supplied by Dr. M. Furue (Kyushu University, School of Medicine, Department of Dermatology). Rabbit polyclonal anti-TGase 1 (NOV-1184-45: NB100-1844 Lot7E3) was purchased from Novus Biologicals (Littleton, CO, USA). Antibodies to phospho-ERK, ERK, phospho-p38, p38, phospho-JNK, JNK, phospho-AKT and AKT were purchased from Cell Signaling Technology (Danvers, MA, USA). Antibodies to phospho-Ser468/Ser276/Ser536NFκBp65, NFκBp65, phospho-Ser133CREB, CREB, c-Jun, phospho-Ser73c-Jun, c-Fos, ATF-2, phospho-Thr71ATF-2, MSK1 and phospho-Thr581/Ser 376/Ser360MSK1 were also purchased from Cell Signaling Technology. Antibodies to COX-2 and β-actin were obtained from Sigma-Aldrich Corp. (St. Louis, MO, USA). The NFκB activation inhibitor II (JSH-23), the MEK inhibitor (PD98059), the p38 inhibitor (SB203580), the JNK inhibitor (JNK inhibitor II) and H89 were purchased from Calbiochem (San Diego, CA, USA). Other chemicals including AX were obtained from Sigma-Aldrich Corp.

### Cell Cultures

HPKs were maintained in Medium 154S supplemented with 5 ng/ml EGF and 50 μg/ml BPE at 37°C with 5% CO_2_. Human HaCaT keratinocytes were maintained in Dulbecco’s modified Eagle medium (DMEM) containing 10% fetal calf serum (FCS), 100 μg/ml penicillin, 100 μg/ml streptomycin and 250 ng/ml amphotericin B at 37°C in a 95% air, 5% CO_2_ atmosphere. In the signaling experiments, HPKs and human HaCaT keratinocytes were cultured in Medium 154S without EGF and BPE and in DMEM without FCS, respectively, after UVB irradiation.

### UVB Irradiation

UVB irradiation was carried out using a SE fluorescent lamp (Clinical Supply, Tokyo, Japan) that provided an energy spectrum with a peak at 305 nm in the UVB region (280–320 nm) as previously reported [24]. The calculation of the emitted dose was performed using a UVB radiometer photodetector (Torex, Tokyo, Japan). Cultured HPKs or human HaCaT keratinocytes in 6 or 10 cm dishes were washed once with phosphate buffered saline (PBS) and were then exposed to UVB irradiation in the presence of PBS. Preliminary testing demonstrated no significant cytotoxic effect on HPKs or human HaCaT keratinocytes by UVB irradiation at 80 mJ/cm^2^ [21, 24] as measured by the MTT assay [25].

### Western Blotting

Immunoblot analysis was performed as previously reported [26, 27]. Briefly, cultured cells were incubated and lysed in lysis buffer (50 mM Tris, pH 7.6, 150 mM NaCl and 1% Triton X-100) containing protease inhibitors (Complete mini-tablets; Boehringer Mannheim, Mannheim, Germany) and/or phosphatase inhibitors (20 mM NaPP, 10 mM NaF and 1 mM Na_3_VO_4_)(only for the signaling experiments) and the lysates were then centrifuged at 13,000 rpm for 15 min. The sediments were solubilized in SDS sample buffer plus 50 mM dithiothreitol (DTT) and were boiled for 5 min. The boiled samples were separated on SDS-PAGE gels and then were transferred onto polyvinylidene difluoride (PVDF) membranes (Bio-Rad, Hercules, CA, USA). After blocking with 5% skim milk in Tris/HCl buffer (TBS, pH 7.5, 100 mM NaCl) containing 0.1% Tween for 1 h, the membranes were treated with monoclonal or polyclonal primary antibodies for 1 h to overnight at room temperature. This was followed by incubation with anti-mouse or anti-rabbit IgG conjugated to horseradish peroxidase for 1 h, and finally, membranes were developed with ECL or ECL plus reagents (Amersham Pharmacia Biotech, Piscataway, NJ, USA). Immunoblots were then exposed to X-ray film for the specified times to detect bands, and specific bands were quantitated with Quantity One (Bio Rad).

### Real-Time RT-PCR Analysis

After UVB exposure, the expression of TGase 1 in HPKs was examined using a real-time reverse transcriptase polymerase chain reaction (RT-PCR) assay and was normalized against glyceraldehyde-3-phosphate dehydrogenase (GAPDH) as previously detailed [28, 29]. Primers with the following sequences were used: GAPDH: Forward 5’-GAAGGTGAAGGTCGGAGTCAACG-3’, Reverse 5’-AGTCCTTCCACGATAACCAAAGTTG-3’, TGase 1: Forward 5’-GCTCGAAGGCTCTGGGTTACA-3’, Reverse 5’-TCGCACAGGCACAAACGAC-3’.

### siRNA Transfection

siRNA transfection was performed with a predesigned siRNA against MSK1 (Silencer Select Pre-designed MSK1 Cat#4392420, Life Technologies Japan, Tokyo, Japan) as described previously [30]. A negative control siRNA (Life Technologies Japan, Tokyo, Japan) was used to monitor for off-target effects. HPKs were cultured in Medium 154S supplemented with 5 ng/ml EGF and 50 μg/ml BPE for 48 h. Transfections were carried out using mixtures of siRNA oligomers and Lipofectamine 2000 (Invitrogen, Melbourne, VIC, Australia) in 50% Opti-MEM I (Invitrogen) and 50% growth medium. An equal volume of growth medium was added at 6 h post-transfection and media were exchanged at 24 h post-transfection. The cells were cultured for a further 24 h and were then exposed to UVB irradiation.

### Miscellaneous Methods

Protein concentrations were determined using a BCA assay kit (Pierce Chemical Co., Rockford, IL, USA) and bovine serum albumin (Amersham Biosciences) as the standard.

### Statistics

All data are expressed as means ± SD (n = 3). For multiple comparisons, data were tested by one-way ANOVA, and subsequently using the Tukey or Dunnet multiple comparison test. P values less than 0.05 are considered statistically significant.

## Results

### Effects of UVB on Gene and Protein Expression Levels of TGase 1

**Fig 1:**
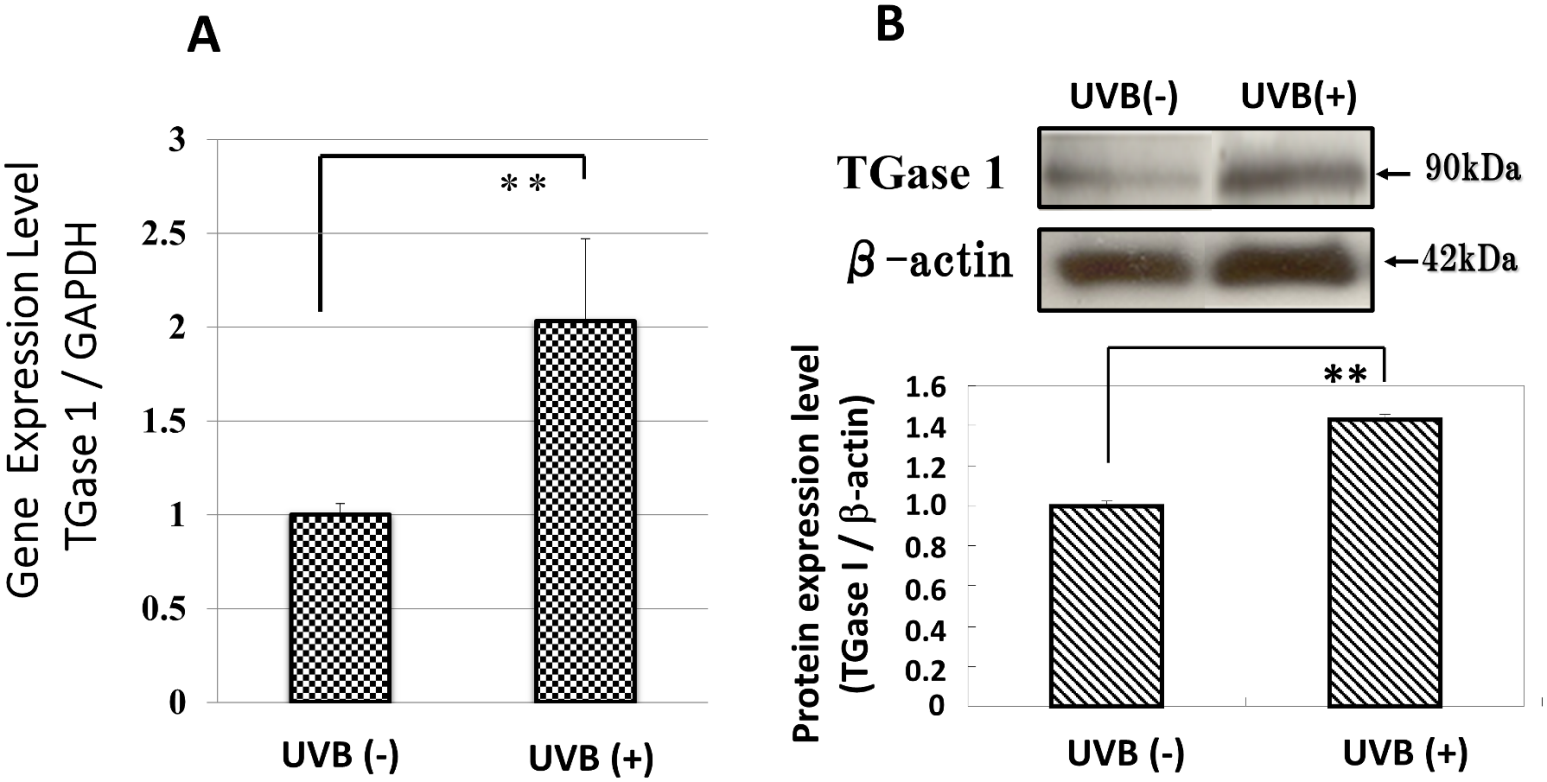
Effects of UVB radiation on the gene and protein expression levels of TGase 1. HPKs were exposed to UVB at a dose of 80 mJ/cm^2^ and were cultured for 24 and 48 h and then analyzed by real-time RT-PCR and Western blotting, respectively. (A) The gene expression level. RT-PCR results are normalized to GAPDH; values are means ± S.D. derived from 3 independent experiments. **: p<0.01. (B) The protein expression level. Representative immunoblots from 3 independent experiments are shown; values are means ± S.D. derived from 3 independent experiments. **: p<0.01.

We first examined the gene and protein expression levels of TGase 1 in HPKs after a single UVB exposure at a dose of 80 mJ/cm^2^. UVB induced a significant increase of TGase 1 at 24 and 48 h post-irradiation at the gene and protein levels, respectively (Fig 1).

### Effects of Stress-Activated Signaling Inhibitors on the UVB-Stimulated Protein Expression of TGase 1

**Fig 2:**
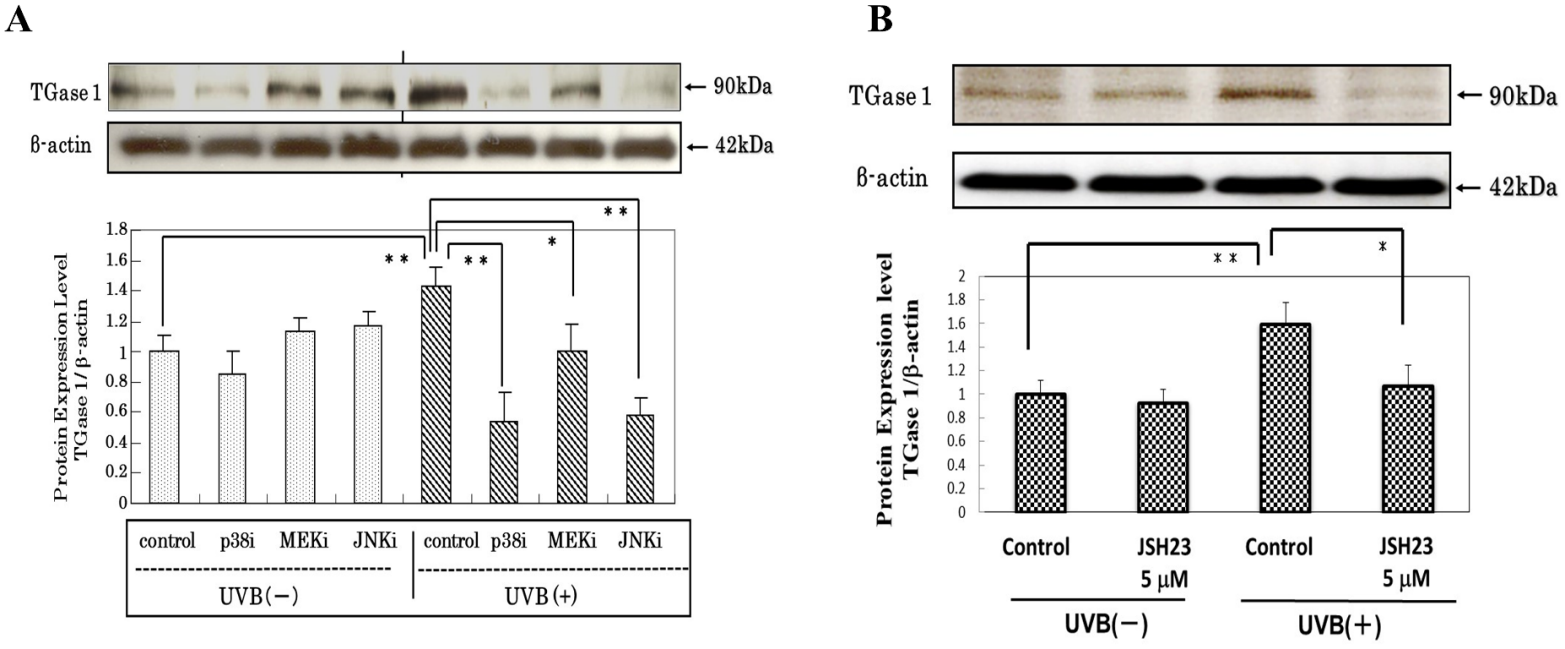
Effects of stress signaling inhibitors on the UVB-stimulated expression of TGase 1 protein. (A) Inhibitors of p38 (at 20 μM), MEK (at 20 μM) and JNK (at 20 μM). (B) Inhibitor of NFκB translocation (at 5 μM). HPKs were incubated with signaling inhibitors as noted for 3 h, then were exposed to UVB at a dose of 80 mJ/cm^2^ and were cultured for 48 h after which cell lysates were subjected to Western blotting analysis. Representative immunoblots from 3 independent experiments are shown; values are means ± S.D. derived from 3 independent experiments. **: p<0.01, *: p<0.05.

When stress-activated signaling inhibitors for p38, MEK and JNK or the NFκB translocation inhibitor JSH23 were added to HPKs 3 h before UVB irradiation and levels of TGase 1 were measured by western blotting at 48 h post-irradiation, increased levels of TGase 1 protein were significantly abrogated by all stress-activated signaling inhibitors tested (Fig 2).

### Effects of AX on the UVB-Stimulated Expression of TGase 1

**Fig 3:**
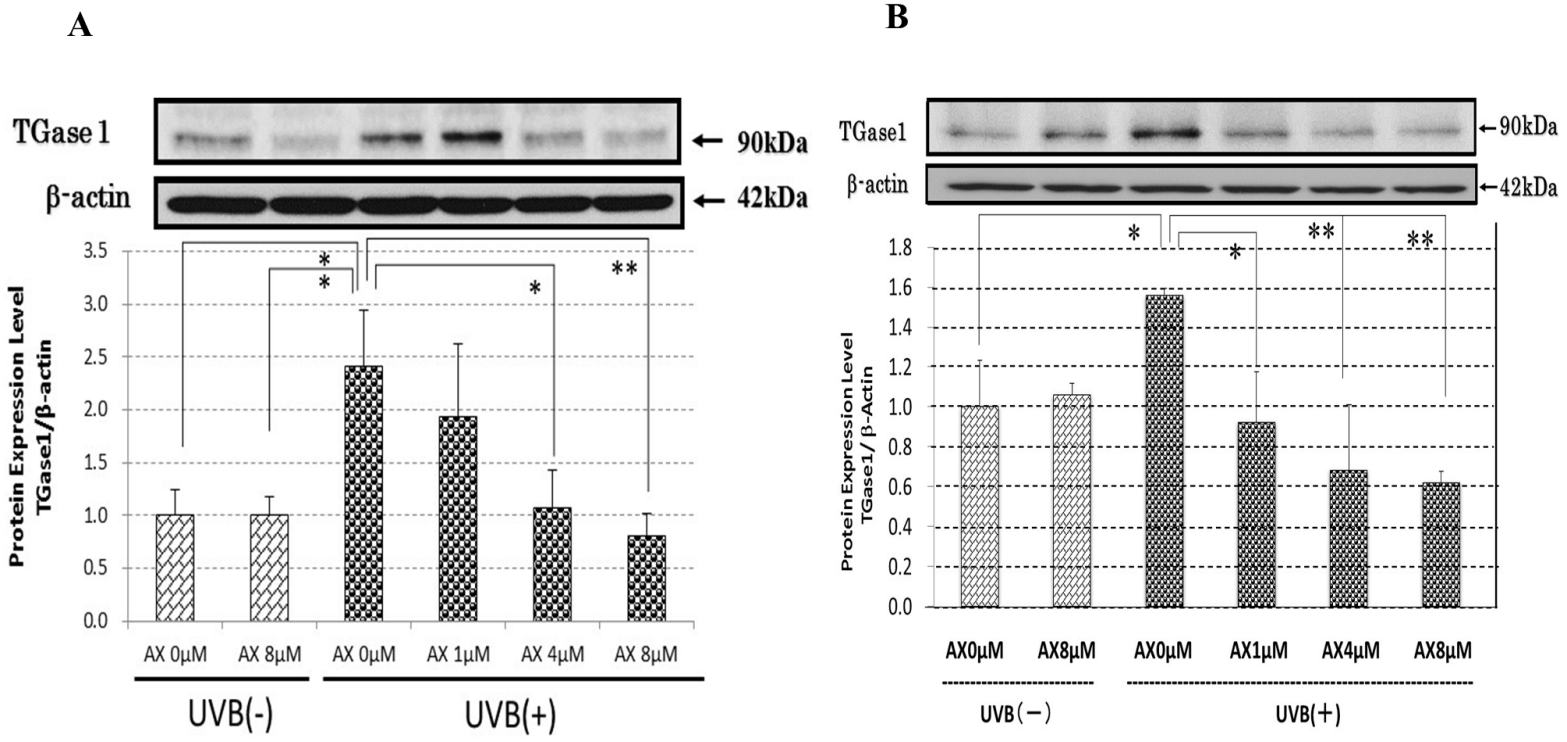
Inhibitory effects of AX on the UVB-stimulated protein expression of TGase 1. (A) HPKs were treated for 3 h with AX at concentrations of 1, 4 or 8 μM, exposed to UVB at a dose of 80 mJ/cm^2^ and were then cultured for 48 h prior to Western blotting analysis. (B) Immediately after UVB irradiation at a dose of 80 mJ/cm^2^, AX was added at concentrations of 1, 4 or 8 μM after which HPKs were cultured for 48 h and subjected to Western blotting analysis. Representative immunoblots from 3 independent experiments are shown; values are means ± S.D. derived from 3 independent experiments. *: p<0.05; **: p<0.01.

When AX was added at a concentration of 1, 4 or 8 μM to HPKs 3 h before UVB exposure at 80 mJ/cm^2^, the increased level of TGase 1 at 48 h post-irradiation was significantly abrogated to the non-irradiated control level at the concentrations of 4 and 8 μM (Fig 3A). Similarly and interestingly, when AX was added at a concentration of 1, 4 or 8 μM to HPKs immediately after UVB exposure at 80 mJ/cm^2^, the increased level of TGase 1 at 48 h post-irradiation was also significantly attenuated at all concentrations tested (Fig 3B).

### Time Course Study of the Phosphorylation of Several Stress-Activated Kinases after UVB-Exposure

**Fig 4:**
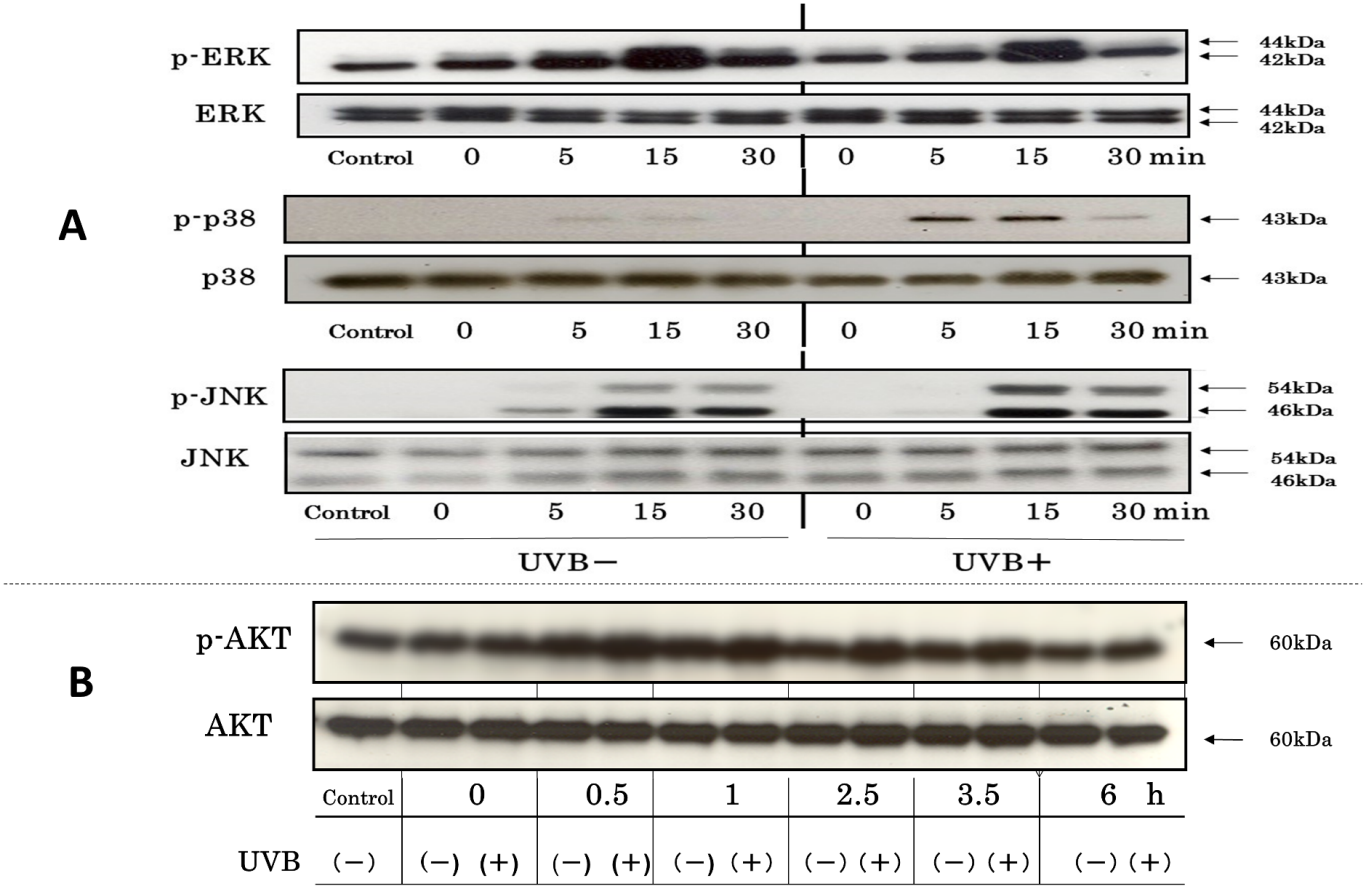
Time course study of the phosphorylation of several stress-activated kinases after UVB exposure. (A) Effects on the phosphorylation of ERK/p38/JNK. (B) Effects on the phosphorylation of AKT. Human HaCaT keratinocytes were exposed to UVB at 80 mJ/cm^2^ and were cultured for the indicated times. Whole cell lysates were prepared and were analyzed by Western blot using antibodies to phospho-p38, p38, phospho-ERK1/2, ERK1/2, phospho-JNK1/2 and JNK1/2, and to phospho-AKT and AKT. Representative data from 3 independent experiments are shown.

The time course study of UVB-exposed human HaCaT keratinocytes revealed an increased phosphorylation of ERK, p38 and JNK with a peak at 15 min after UVB exposure. It should be noted that only an exchange from PBS (the irradiation buffer) to fresh signaling medium without UVB radiation also increased the phosphorylation of ERK and JNK with a peak at 15 min post-medium exchange, to a similar or a slightly lesser extent compared to the increased level of ERK or JNK phosphorylation, respectively, at 15 min post-irradiation in UVB-exposed cells (Fig 4A). This indicates that UVB radiation itself does not stimulate ERK phosphorylation or can slightly increase it, depending on the experimental conditions. In contrast, UVB radiation elicited a prolonged and sustained phosphorylation of AKT with an increase starting at 15 min post-irradiation and persisting until at least 6 h post-irradiation (Fig 4B).

### Effects of AX on the UVB-Induced Phosphorylation of p38/ERK/JNK/c-Jun/ATF-2 and on the UVB-Induced Protein Expression of c-Jun and c-Fos

**Fig 5:**
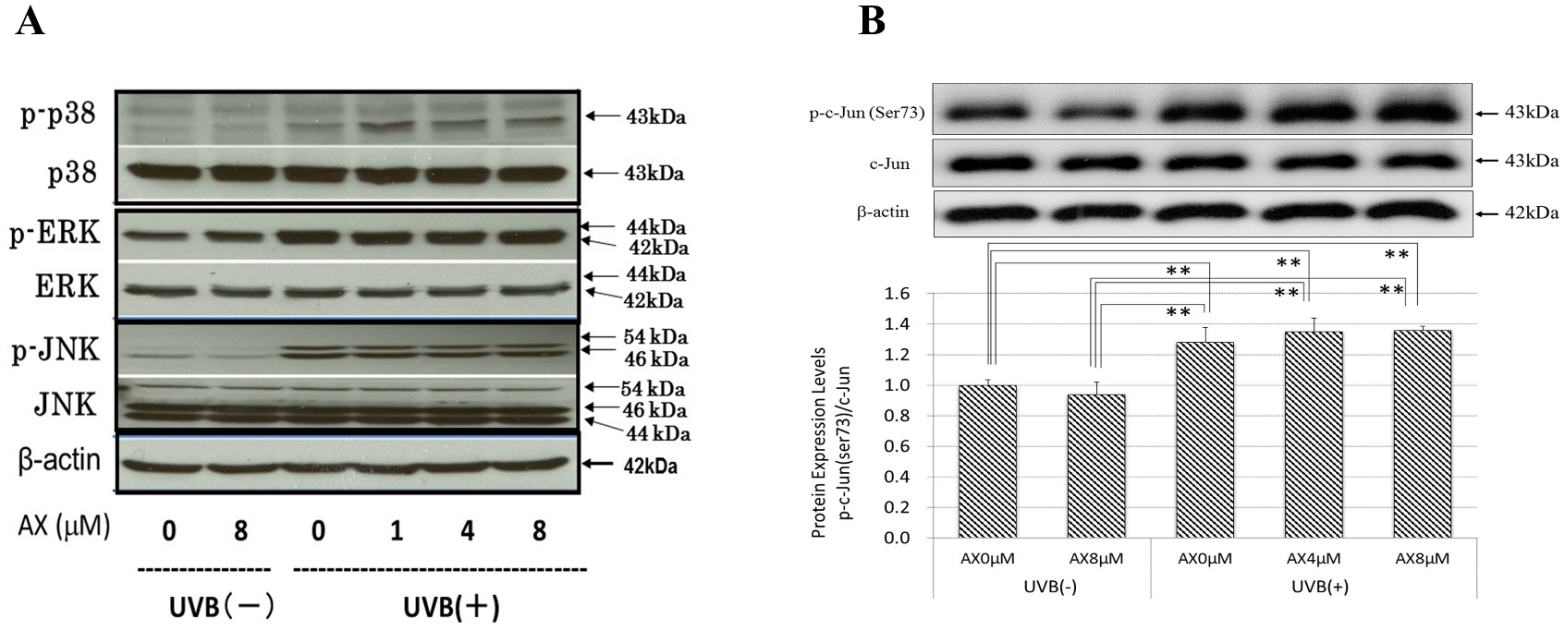
Effects of AX on the UVB-induced phosphorylation of p38/ERK/JNK/c-Jun. (A) Effects of post-irradiation treatment with AX on the UVB-induced phosphorylation of p38, JNK and ERK in human HaCaT keratinocytes. (B) Post-irradiation treatment for the phosphorylation of c-Jun in HPKs. HPKs or human HaCaT keratinocytes were exposed to UVB at a dose of 80 mJ/cm^2^ immediately after which the cells were treated with AX at the indicated concentrations. Lysates were harvested at the indicated times after UVB irradiation and were immunoblotted with antibodies to phosphorylated or non-phosphorylated p38/ERK/JNK/c-Jun. Representative immunoblots from 3 independent experiments are shown; values are means ± S.D. from 3 independent experiments. **: p<0.01; *: p<0.05.

**Fig 6:**
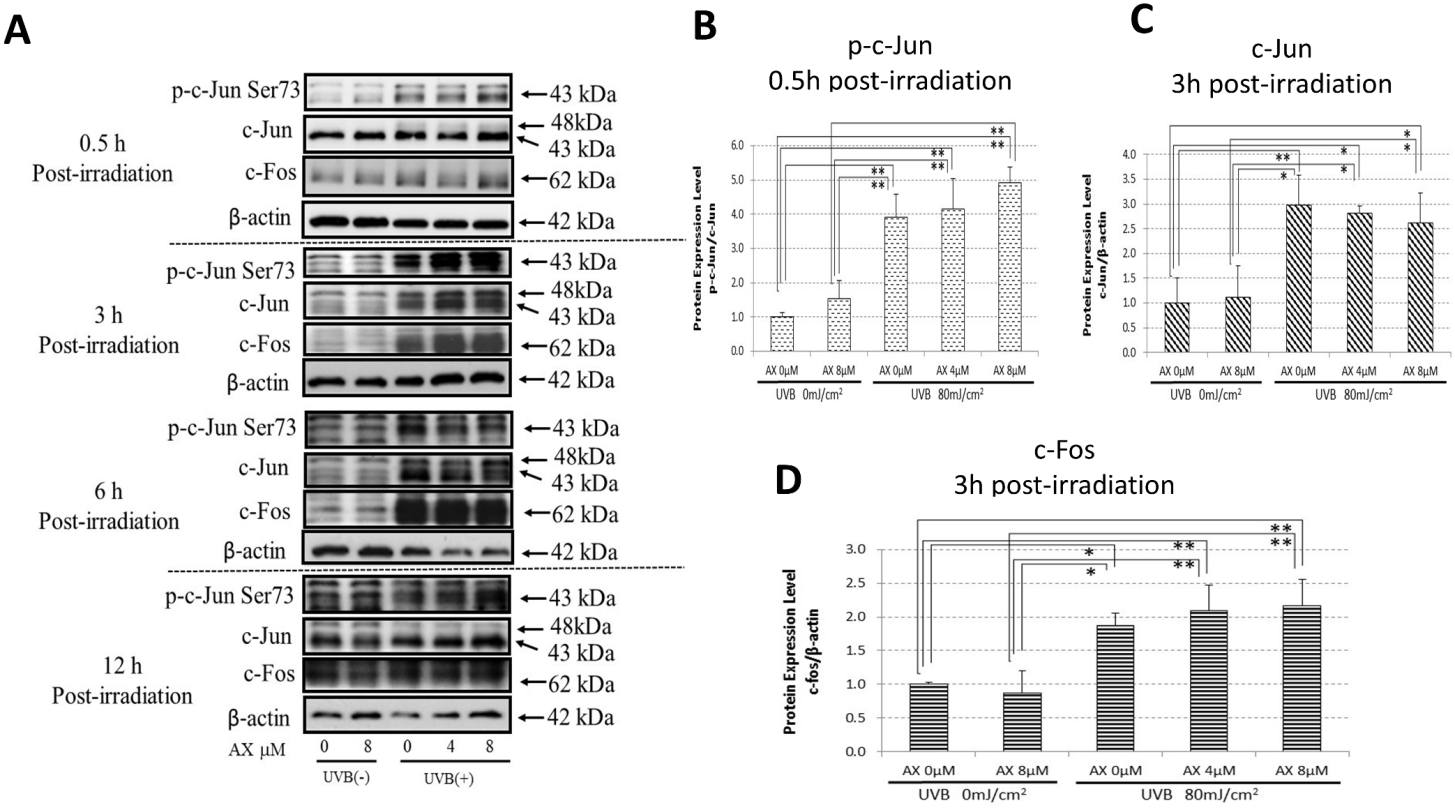
Effects of AX on the UVB-induced phosphorylation of c-Jun and on the UVB-induced protein expression of c-Jun and c-Fos. (A) Western blotting for the phosphorylation of c-Jun and the protein expression of c-Jun and c-Fos. (B) Densitometric analysis for the phosphorylation of c-Jun at 0.5 h post-irradiation. (C) Densitometric analysis for the protein level of c-Jun at 3 h post-irradiation. (D) Densitometric analysis for the protein level of c-Fos at 3 h post-irradiation. Human HaCaT keratinocytes were exposed to UVB at a dose of 80 mJ/cm^2^ immediately after which the cells were treated with AX at the indicated concentrations. Lysates were harvested at the indicated times after UVB irradiation and were immunoblotted with antibodies to phosphorylated or non-phosphorylated c-Jun or c-Fos. Representative immunoblots from 3 independent experiments are shown; values are means ± S.D. from 3 independent experiments. **: p<0.01; *: p<0.05.

**Fig 7:**
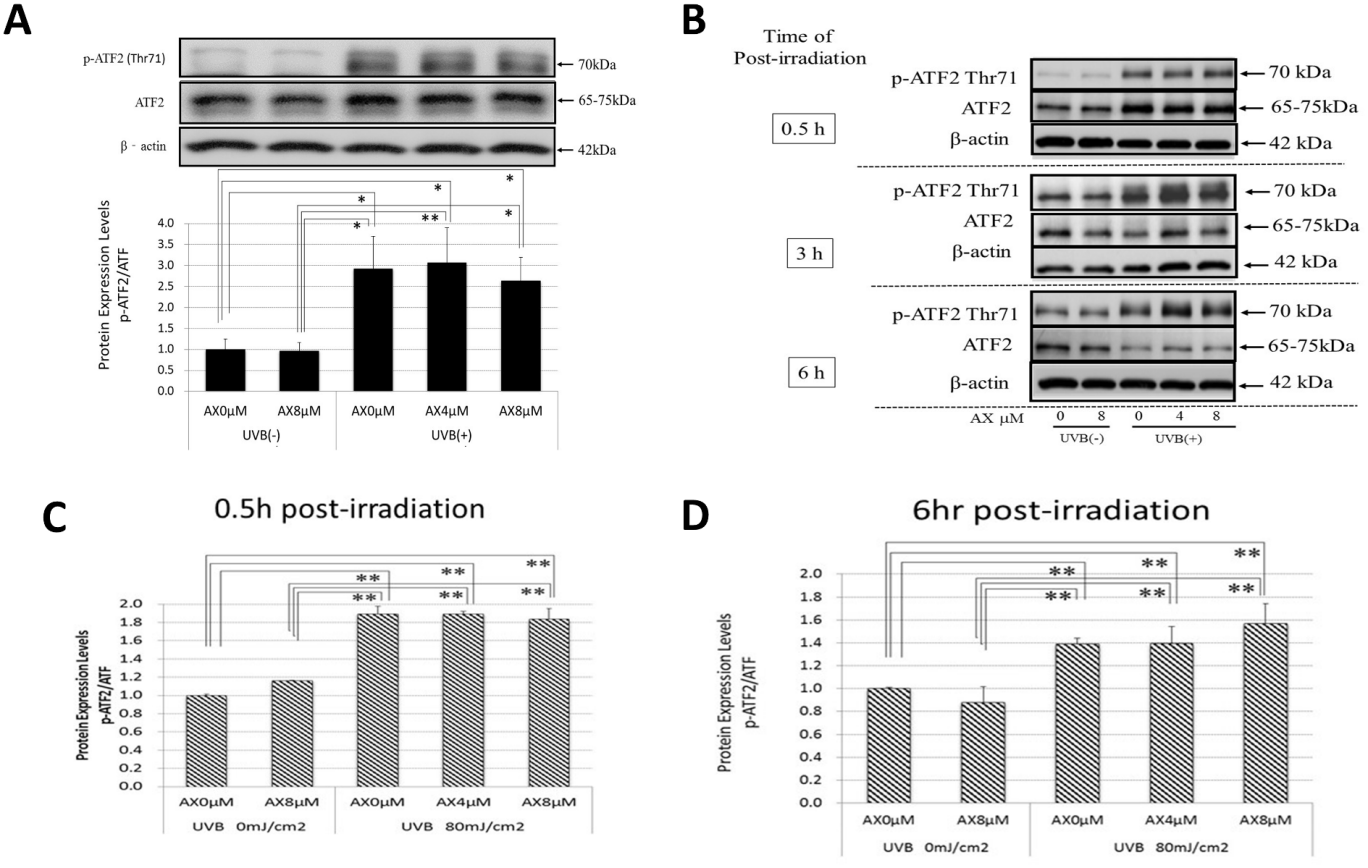
Effects of AX on the UVB-induced phosphorylation of ATF2. (A) Western blotting for the phosphorylation of ATF-2 in HPKs. (B) Western blotting for the phosphorylation of ATF-2in human HaCaT keratinocytes. (C) Densitometric analysis for phosphorylation of ATF-2 at 0.5 h post-irradiation. (D) Densitometric analysis for the phosphorylation of ATF-2 at 6 h post-irradiation. HPKs or human HaCaT keratinocytes were exposed to UVB at a dose of 80 mJ/cm^2^ immediately after which the cells were treated with AX at the indicated concentrations. Lysates were harvested at the indicated times after UVB irradiation and were immunoblotted with antibodies to phosphorylated or non-phosphorylated ATF-2. Representative immunoblots from 3 independent experiments are shown; values are means ± S.D. from 3 independent experiments. **: p<0.01.

We have already reported that AX distinctly abolishes the increased phosphorylation of p38 and ERK at 15 min post-irradiation in UVB-exposed HPKs when treated pre-irradiation [21]. On the other hand, the addition of AX immediately after UVB radiation does not abrogate the increased phosphorylation of the same signaling molecules at 15 min post-irradiation in UVB-exposed HPKs [21]. Similarly, when human HaCaT keratinocytes were exposed to UVB at a dose of 80 mJ/cm^2^, the pre- (data not shown) but not post-irradiation treatment with AX distinctly abolished the increased phosphorylation of p38, JNK and ERK at 15 min post-irradiation in UVB-exposed human HaCaT keratinocytes (Fig 5A), which resembles the effects observed for HPKs. Similarly, while the Ser73 or Ser71 phosphorylations of c-Jun or ATF-2 and the protein expression of c-Fos/c-Jun were markedly increased in UVB-exposed HPKs or human HaCaT keratinocytes at 0.5 and 3 h post-irradiation, respectively, the significant increase of Ser73 or Ser71 phosphorylation of c-Jun or ATF-2 and the increased protein expression of c-Fos/c-Jun were not abrogated by the post-irradiation treatment with AX at 0.5–3 h and 3–12 h post-irradiation, respectively (Figs 5B, 6 and 7).

### Effects of AX on the UVB-Induced Phosphorylation of CK2 and the Degradation of I-κBα

**Fig 8:**
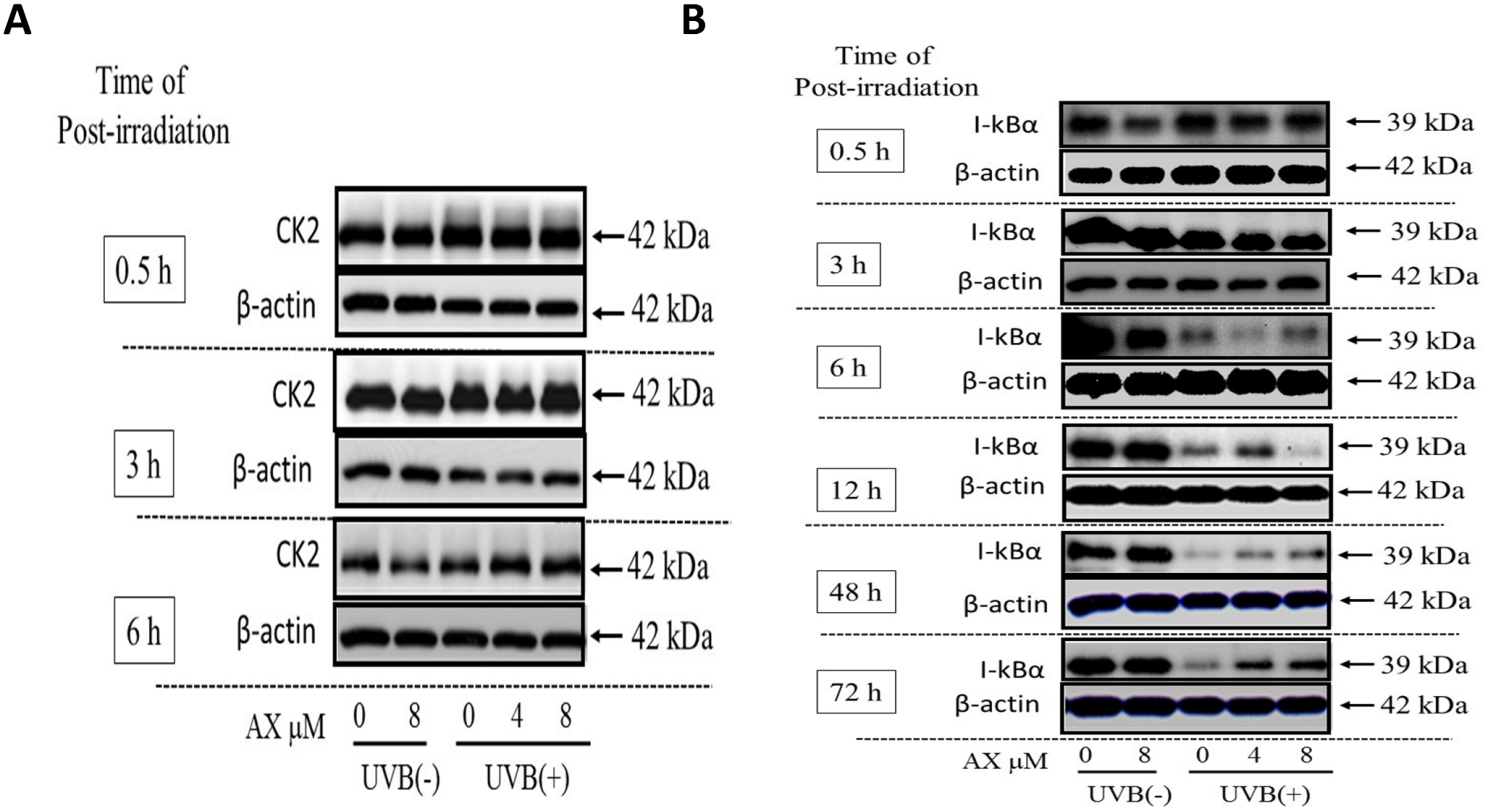
Effects of AX on the UVB-induced phosphorylation of CK2 and the degradation of I-κBα. (A) CK2, (B) I-κBα. Human HaCaT keratinocytes were exposed to UVB at a dose of 80 mJ/cm^2^ immediately after which the cells were treated with AX at the indicated concentrations. Lysates were harvested at the indicated times after UVB irradiation and were immunoblotted with antibodies to CK2 or I-κBα. Representative immunoblots from 3 independent experiments are shown.

When human HaCaT keratinocytes were exposed to UVB at a dose of 80 mJ/cm^2^, the phosphorylation of CK2 (as detected by the appearance of bands with increased molecular mass) and the protein levels of I-κBα were distinctly increased and markedly decreased at 30 min and 6 h post-irradiation, respectively, (Fig 8). When AX was added to human HaCaT keratinocytes at a concentration of 4 or 8 μM immediately after UVB exposure (at 80 mJ/cm^2^), the slight shift of CK2 protein toward a higher molecular mass and the decreased level of I-κBα protein was not abrogated at 0.5–3.0 h and 6–48 h post-irradiation, respectively (Fig 8).

### Effects of AX on the Ser276/Ser468/Ser536 Phosphorylation of NFκBp65 in UVB-Exposed Human Keratinocytes

**Fig 9:**
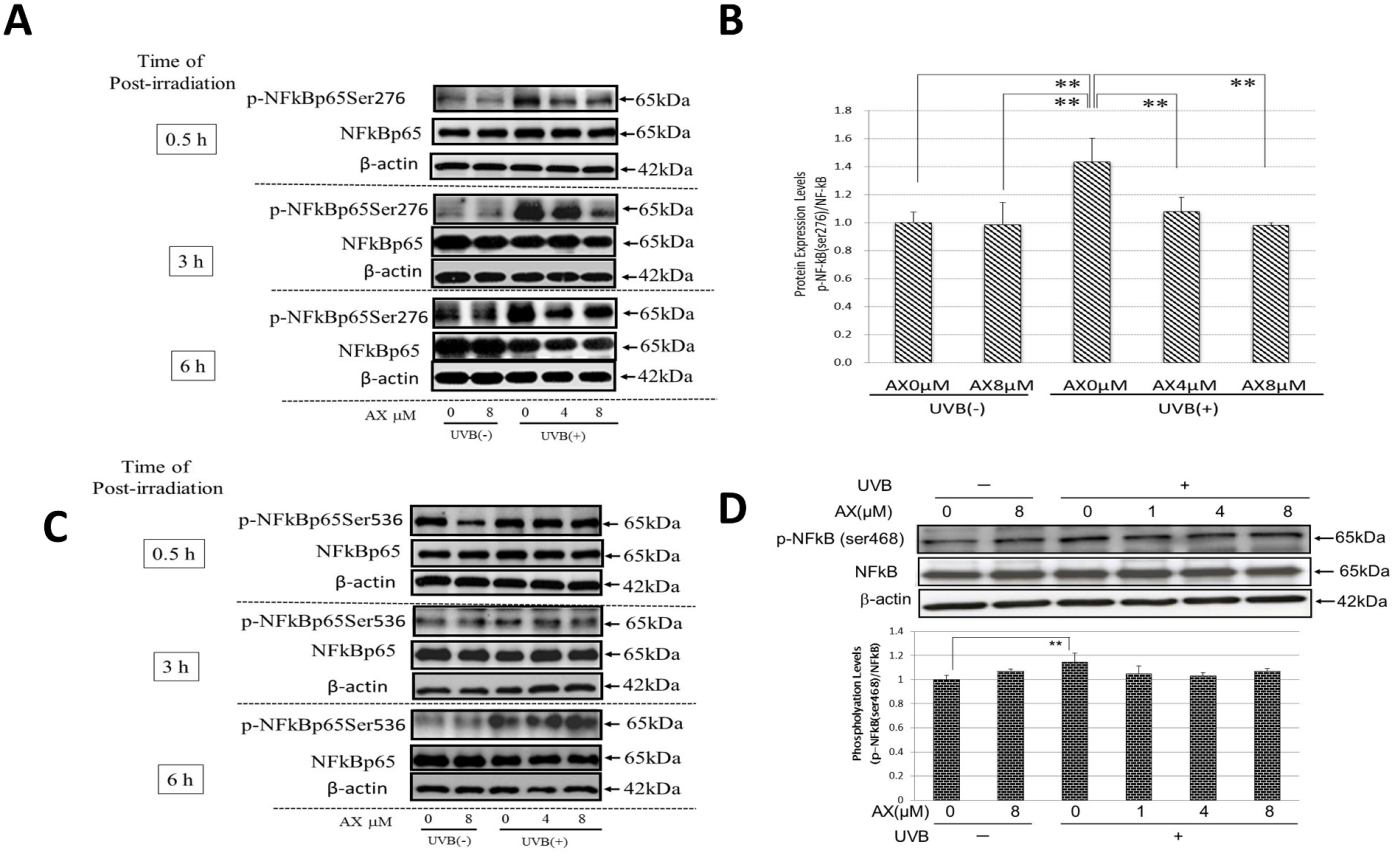
Effects of AX on the Ser276/468/536 phosphorylations of NFκBp65 in UVB-exposed human HaCaT keratinocytes. (A) Ser276 phosphorylation of NFκBp65 at 0.5, 3 and 6 h post-irradiation. (B) Densitometric analysis for Ser276 phosphorylation of NFκBp65 at 0.5 h post-irradiation. (C) Ser536 phosphorylation of NFκBp65 at 0.5, 3 and 6 h post-irradiation. (D) Ser468 phosphorylation of NFκBp65 at 0.5 h post-irradiation. Human HaCaT keratinocytes were exposed to UVB irradiation at a dose of 80 mJ/cm^2^ immediately after which the cells were treated with AX at the indicated concentrations. Lysates were harvested at 0.5, 3 or 6 h after UVB irradiation and were immunoblotted with antibodies to NFκBp65 and phosphorylated NFκBp65Ser276/Ser536/Ser468. Representative immunoblots from 3 independent experiments are shown; values are means ± S.D. from 3 independent experiments. **: p<0.01.

When human HaCaT keratinocytes were exposed to UVB at a dose of 80 mJ/cm^2^, the Ser276, Ser468 or Ser536 phosphorylations of NFκBp65 were markedly increased at 0.5–6 h post-irradiation (Fig 9). When AX was added to human HaCaT keratinocytes at a concentration of 1, 4 or 8 μM immediately after UVB exposure (80 mJ/cm^2^), the significant increase of the Ser276 but not the Ser468/Ser536 phosphorylation of NFκBp65 was significantly abolished at 0.5–6.0 h post-irradiation (Fig 9).

### Effects of UVB Irradiation on the Thr581/Ser376/Ser360 Phosphorylation of MSK1 and the Inhibitory Effect of AX

**Fig 10:**
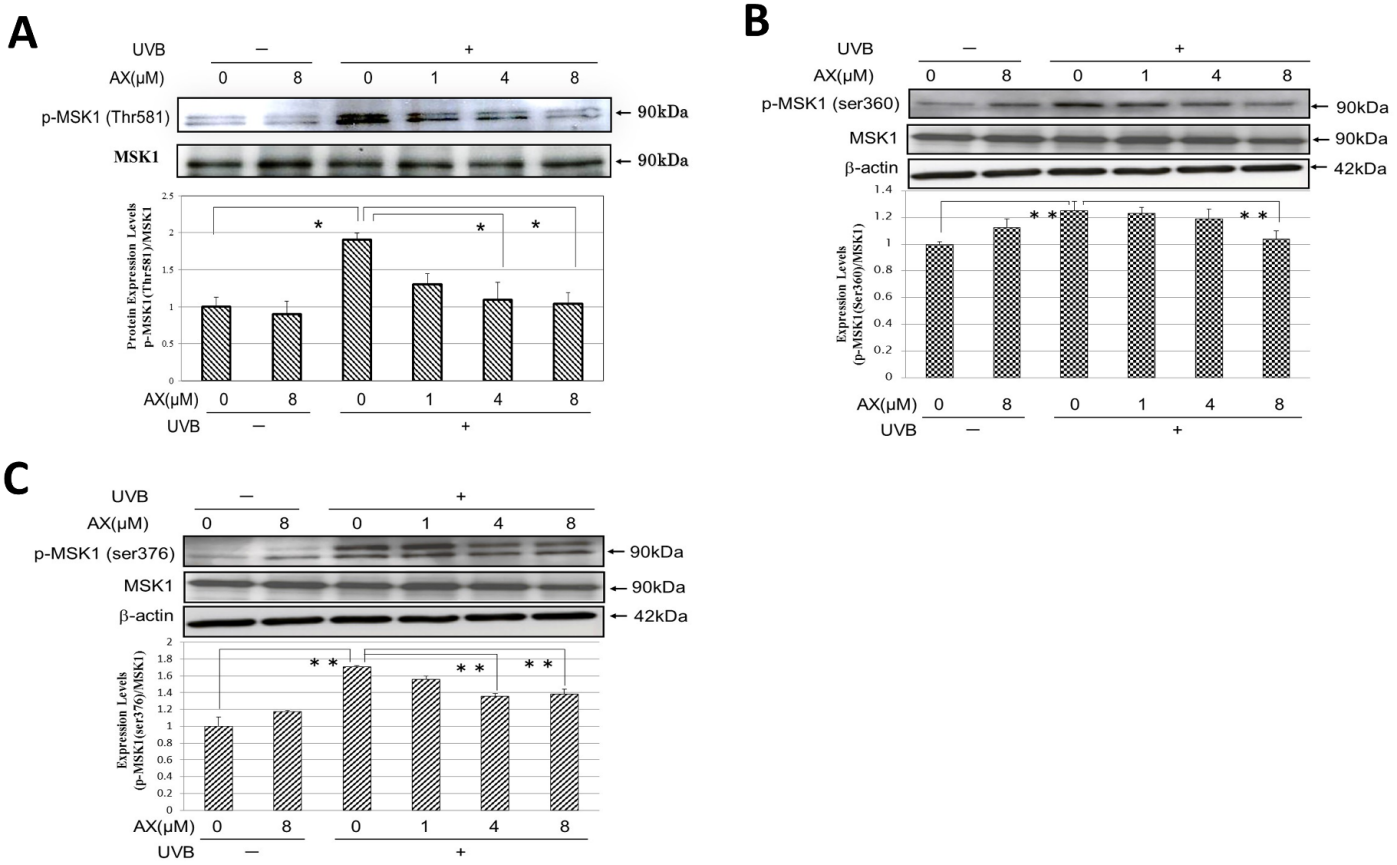
Effects of UVB irradiation on the Thr581/Ser360/Ser376 phosphorylation of MSK1 and the inhibitory effect of AX. (A) Thr581 phosphorylation of MSK1 in HPKs at 0.5 h post-irradiation. (B), (C) Ser360/ Ser376 phosphorylation of MSK1 in human HaCaT keratinocytes at 0.5 h post-irradiation. HPKs or human HaCaT keratinocytes were exposed to UVB irradiation at a dose of 80 mJ/cm^2^ immediately after which the cells were treated with AX at the indicated concentrations. Lysates were harvested at 30 min after UVB irradiation and were immunoblotted with antibodies to Thr581/Ser376/360 phosphorylated MSK1. Representative immunoblots from 3 independent experiments are shown; values are means ± S.D. from 3 independent experiments. *: p<0.05, **:p<0.01.

In the present study, we found for the first time that UVB exposure (80 mJ/cm^2^) of HPKs significantly stimulated the Thr581 phosphorylation of MSK1 at 0.5 h post-irradiation, which was significantly abrogated by treatment with AX at 4 or 8 μM following UVB irradiation (Fig 10A). In UVB-exposed human HaCaT keratinocytes, while the Ser360/Ser376 phosphorylation of MSK1 was remarkably increased at 0.5 h post-irradiation, the post-irradiation treatment with AX distinctly abrogated those increases (Fig 10B and 10C).

### Effects of AX on the Ser133 Phosphorylation of CREB in UVB-Exposed Human Keratinocytes

**Fig 11:**
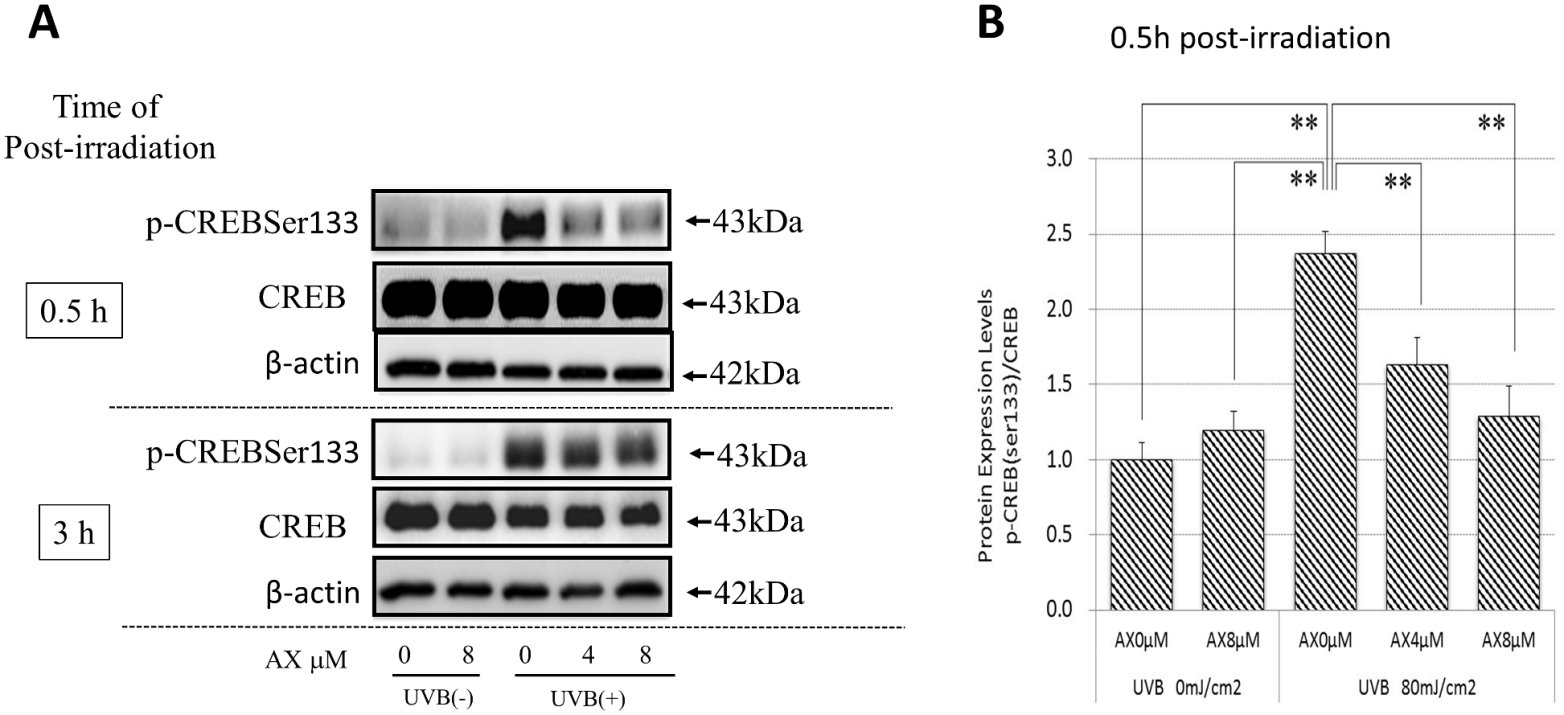
Effects of AX on the Ser133 phosphorylation of CREB in UVB-exposed human HaCaT keratinocytes at 0.5 and 3 h min post-irradiation. (A) Western blotting for the phosphorylation of CREB Ser133. (B) Densitometric analysis for Ser133 phosphorylation of CREB at 0.5 h post-irradiation. Human HaCaT keratinocytes were exposed to UVB irradiation at a dose of 80 mJ/cm^2^ immediately after which they were treated with AX at the indicated concentrations. Lysates were harvested at 0.5 and 3 h after UVB irradiation and were immunoblotted with antibodies to phosphorylated or non-phosphorylated *CREB*. Representative immunoblots from 3 independent experiments are shown; values are means ± S.D. from 3 independent experiments. **: p<0.01.

When human HaCaT keratinocytes were exposed to UVB at a dose of 80 mJ/cm^2^, the Ser133 phosphorylation of CREB was markedly increased at 0.5–6 h post-irradiation (Fig 11). When AX was added to human HaCaT keratinocytes at concentrations of 4 or 8μM immediately after UVB exposure (80 mJ/cm^2^), the significant increase of Ser133 phosphorylation of CREB was significantly abolished at 0.5 h post-irradiation, the abrogation of which was sustained until 3 h post-irradiation (Fig 11).

### Effects of the MSK1 Inhibitor H89 on the Increased Phosphorylation of NFκBp65Ser276 and CREBSer133 as well as on the Protein Expression of TGase 1 and COX-2 in UVB-Exposed Human Keratinocytes

**Fig 12:**
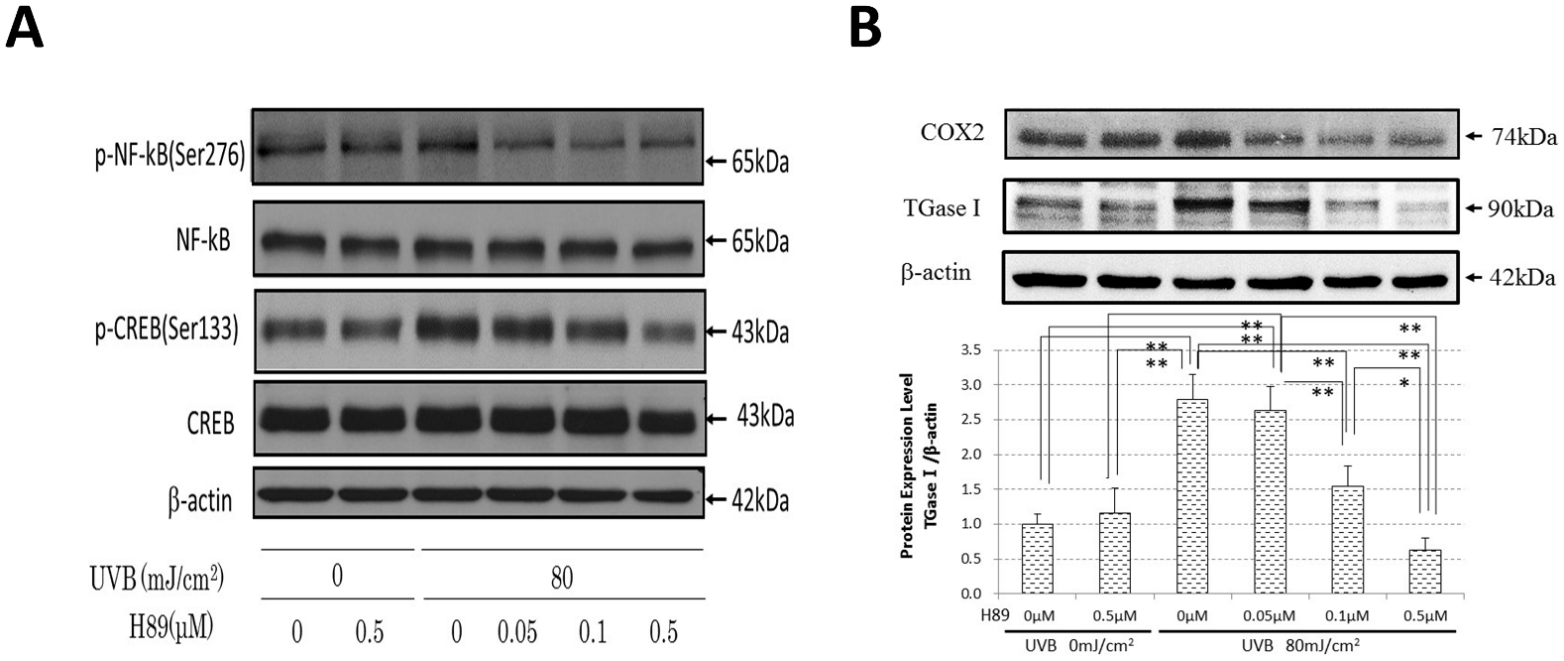
Effects of the MSK1 inhibitor H89 on the increased phosphorylation of NFκBp65Ser276 and CREBSer133 as well as on the protein expression of TGase 1 and COX-2 in UVB-exposed human keratinocytes. (A) Effects on the phosphorylation of NFκBp65Ser276 and CREBSer133. (B) Effects on the protein expression of TGase 1 and COX-**2**. HPKs were incubated with H89 at the indicated concentrations immediately after UVB radiation at a dose of 80 mJ/cm^2^ and were subsequently cultured for 0.5 h for the phosphorylation analysis or 48 h for the protein expression analysis prior to Western blotting. Representative immunoblots from 3 independent experiments are shown; values are means ± S.D. derived from 3 independent experiments. ** p < 0.01, * p<0.05.

Western blot analysis demonstrated that the post-irradiation treatment with the MSK1 inhibitor H89 down-regulated the increased phosphorylation of NFαBp65Ser276 and CREBSer133 but not Thr565/Ser376/360MSK1 (data not shown) at concentrations of 0.5 and 0.05–0.5 μM, respectively, at 0.5 h post-irradiation in UVB-exposed HPKs (Fig 12A). Treatment with H89 also significantly abrogated the increased protein expression of TGase 1 and COX-2 at 48 h post-irradiation in a dose-dependent manner at concentrations of 0.1 and 0.5 μM (Fig 12B).

### Effects of Silencing of MSK1 mRNA on the UVB-Stimulated Expression of TGase 1

**Fig 13:**
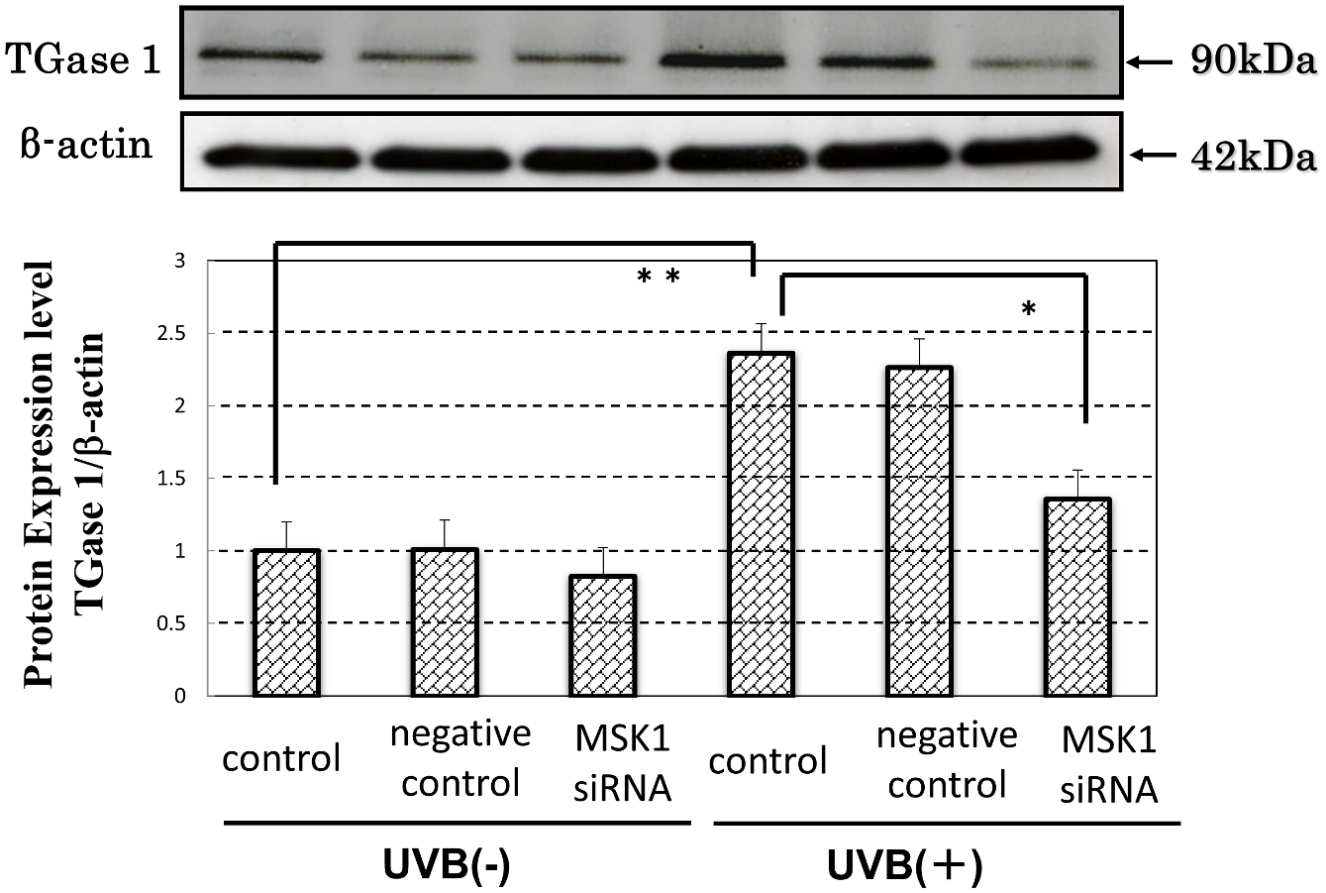
Effects of silencing of MSK1 mRNA on the UVB-stimulated expression of TGase 1 protein. HPKs were transfected with a pre-designed siRNA against MSK1 to down-regulate MSK1 along with a negative control siRNA. Twenty-four h after transfection, HPKs were exposed to UVB irradiation at a dose of 80 mJ/cm^2^ and were cultured for 48 h after which whole cell lysates were prepared for Western blot analysis using antibodies to TGase 1 and β-actin. Representative immunoblots from 3 independent experiments are shown; values are means ± S.D. derived from 3 independent experiments. *: p<0.05; **: p<0.01.

Transfection of MSK1 siRNA in HPKs significantly reduced the UVB-stimulated expression of TGase 1 protein without any changes in unexposed HPKs (Fig 13).

## Discussion

The present study demonstrates for the first time that UVB exposure of human keratinocytes significantly up-regulates the expression of TGase 1 at both the transcriptional and translational levels. This suggests that the stimulated expression of TGase 1 is highly involved in the accentuated keratinization and desquamation which occurs in UVB-exposed human skin. Our study also shows for the first time the detailed evidence on multiple UVB-activated signaling cascades that are the front lines of stress-activated signaling, such as p38, ERK and JNK, and are distinctly activated (phosphorylated) with a peak at 15 min post-irradiation in UVB-exposed human keratinocytes. Further, the results show that those downstream signaling pathways, including NFκB, IKK, CK-2/I-κBα, AP-1(c-Fos/c-Jun), ATF-2 and MSK1, are also subsequently activated. Therefore, we used several inhibitors of those stress-activated signaling factors to elucidate the intracellular signaling mechanism(s) involved in the UVB-induced accentuation of TGase 1 expression. We found that the UVB-induced increase of TGase 1 is significantly abrogated by inhibitors of the front lines of stress-activated signaling, p38, ERK (to a lesser extent) and JNK, when added prior to the UVB irradiation of human keratinocytes. This suggests that those stress-activated signaling factors are involved in the UVB-induced accentuation of TGase 1 expression.

UVB exposure of keratinocytes is well known to activate NFκB signaling by stimulating IKKkinase, which phosphorylates IκκBα, enabling NFκBp65 to release toward translocation into nuclei during the signaling pathway downstream of p38 or ERK activation [31] and/or via an autocrine loop by secreted IL-1α [32]. Therefore, we next used the NFκB translocation inhibitor JSH23 to clarify the association of NFκB signaling downstream of those stress-activated signaling factors with TGase 1 expression. The up-regulated expression of TGase 1 elicited by UVB was significantly abrogated by pre-irradiation treatment with JSH23, which suggests that NFκB signaling, including its translocation into nuclei downstream of p38, ERK or JNK, or of IL-1α triggered signaling in an autocrine fashion, is substantially involved in the UVB-induced accentuation of TGase 1 expression.

Many studies have reported that AX [33, 34] as well as other antioxidants [35, 36, 37, 38] distinctly attenuate the NFκB pathway by suppressing the activation of IKKinase, which results in the diminished phosphorylation of I-κB, in turn leading to the abolishment of the separation of NFκB from I-κB. This finally interrupts the nuclear translocation of NFκB. However, all those studies observed the intracellular effects of pretreatment with AX or other antioxidants in stimulant-treated cells. We have already reported that AX has differential effects on UVB-induced activation of stress-activated signaling pathways when treated before or after UVB radiation in HPKs and that post-irradiation treatment affects the UVB-stimulated phosphorylation of MSK1Ser365 (the auto-phosphorylation site subsequent to the phosphorylation at Ser360 and Thr581 [39, 40, 41]) without an inhibitory effect on the front line of stress-activated signaling pathways in HPKs [21]. It remains to be elucidated whether other multiple signaling molecules, at least including MSK1Thr582/Ser360 (phosphorylation sites by p38 and ERK, respectively, or both [39, 40, 41]), downstream of the front line of stress-activated signaling pathways are also affected. Our observation that the UVB-accentuated expression of TGase 1 is significant abrogated by AX treatment post-irradiation will provide a deep insight into understanding the detailed signaling mechanisms involved once the inhibition signaling sites downstream of the front line of stress-activated signaling pathways by the post-irradiation treatment with AX are defined in detail.

In this approach, consistent with our previous study in HPKs [21], AX interrupts ROS-mediated stress-activated signaling such as p38, ERK and JNK when treated pre-irradiation whereas the post-irradiation treatment with AX cannot inhibit the phosphorylation of those signaling molecules in UVB-exposed human HaCaT keratinocytes. We have also found for the first time that there was no abrogating effect of the post-irradiation treatment with AX on the accentuated phosphorylation of ATF-2 (Ser71) or c-Jun (Ser73) and the increased protein expression of c-Fos/c-Jun, signaling lineages downstream of p38 (for p-ATF-2 [42]), JNK (for p-Thr-71ATF-2 [43] and p-c-Jun/c-Fos [44]] and ERK (for p-Thr-71ATF-2 [43] and c-Jun [45, 46]), indicating there is no involvement of the ATF-2 and AP-1 pathways.

CK2 is a c-terminal I-κB kinase responsible for degradation of I-κB**α**, which is involved in NFκB activation during the UV response and CK2 activity is UV-inducible through a mechanism that depends on the activation of p38 MAPK [47]. In this study, we found for the first time that the UVB-induced activation of the signaling lineages for the CK2/I-κBα/NFκB axis downstream of p38 [47] is not abrogated by the post-irradiation treatment with AX, which suggests no association of the inhibitory effect of AX with the mainstream of the NFκB pathway leading, through the degradation of I-κBα, to the translocation of NFκBp65 into nuclei. This is in agreement with our previous confocal microscopic study [21] that showed no inhibitory effect of AX post-irradiation on the translocation of NFαB into nuclei. Consistently, Ser536 phosphorylation of NFκBp65 occurring prior to that nuclear translocation in association with the phosphorylation and subsequent degradation of I-κBα, all of which occur downstream of IKKinase activation, was distinctly accentuated in UVB-exposed human HaCaT keratinocytes, but that phosphorylation was not abrogated by the same treatment with AX. Further, the increased phosphorylation of Ser468 of NFκBp65, which occurs downstream of the phosphatidylinositol 3-kinase (PI3K)/Akt/GSK3β pathway [48], was not abrogated in human HaCaT keratinocytes by the post-irradiation treatment with AX. These results suggest that the p38/CK-2/I-κBα/NFκBp65Ser536 axis, the ERK/IKK/I-κBα/NFκBp65Ser536 axis and the phosphatidylinositol 3-kinase (PI3K)/Akt/GSK3β/NFκBp65Ser468 axis are not involved in the inhibitory effect of the post-irradiation treatment with AX on the increased expression of TGase 1.

During a search for the interrupted signaling lineages by the post-irradiation treatment with AX, which is consistent with our previous study in HPKs [21], we finally found that the UVB-induced increase of Ser276 phosphorylation of NFκBp65, which occurs downstream of MSK1 within the nuclei after the translocation of NFκBp65, was significantly abolished by AX even when treated post-irradiation in UVB-exposed human HaCaT keratinocytes.

In epidermal keratinocytes, MSK1 is phosphorylated at either Ser360 or Thr581 by p38 MAPK or by ERK p44/42 MAPKs [39, 40, 41] and that phosphorylation at Ser360 is an essential requirement for MSK activation [49]. Further, Ser376 is auto-phosphorylated subsequent to the phosphorylation at Ser360 and Thr581 [39, 40, 41]. The activated MSK1 phosphorylates NFκBp65 at Ser276 after translocation into nuclei, which results in the enhancement of its DNA binding activity and subsequently of its transcriptional activity [50, 51, 52, 53]. Thus, it is anticipated that the inhibition of MSK1 activation leads to the inhibition of Ser276 phosphorylation of translocated NFκBp65 in nuclei, which down-regulates the binding activity of NFκBp65 with targeted DNA sequences, resulting in a reduced transcriptional activity. In this study, we have found for the first time that the observed inhibition of Ser276 phosphorylation of NFκBp65 is accompanied by the abrogating effect on the Thr581 and subsequently Ser360/Ser376 phosphorylation of MSK1 that occurs downstream of either p38 and/or ERK activation [39]. Since Thr581 of MSK1 is the initial phosphorylation site by activated p38 MAPK or ERK [39, 40], it is likely that the observed inhibition of auto-phosphorylation of MSK1 at Ser376 by the post-irradiation treatment with AX results from the inhibition of phosphorylation of MSK1 at Thr581. Further, the inhibitory effect of AX on MSK1 activation is substantiated by our observations that the Ser133 phosphorylation of CREB, which occurs downstream of activated MSK1, was also abrogated by the post-irradiation treatment with AX. Thus, the mode of action by AX indicates that the post-irradiation treatment with AX does not affect the activation of p38 MAPK as well as the signaling pathways of the JNK/c-Jun/AP-1 axis, the p38/ATF-2 axis and the p38/CK-2/I-κBα/NFκBp65Ser536 axis (including the translocation of NFκB into nuclei). However, the same treatment specifically triggers p38/MSK1 association to inhibit p38 MAPK activity in a MSK1-dependent manner, which leads to the specific interruption of the signaling pathway of the p38/MSK1/translocated NFκBp65Ser276/CREB axis.

Available evidence indicates that TGase 1 expression in keratinocytes is regulated primarily by activator protein 1 (AP-1), which consists of heterodimers between members of the Jun and Fos families of transcription factors, such as c-Jun and c-Fos [54, 55, 56, 57], the activation of which occurs predominantly through the phosphorylation of c-Jun by upstream JNK activation. In human HaCaT keratinocytes, UVB irradiation has been shown to up-regulate AP-1 by increasing the expression of c-Fos via the activation of CREB [58]. Our observation that a JNK inhibitor suppresses the up-regulated protein levels of TGase 1 in UVB-exposed human keratinocytes is in agreement with the involvement of AP-1. However, to our knowledge, there have been no reports describing the involvement of the NFκB signaling pathway in TGase 1 expression.

Our observations on the effects of AX on the UVB-induced activation of stress-activated signaling pathways strongly suggest that the abrogation of MSK1 activation is the only and initial inhibitory site affected by the post-irradiation treatment with AX, which leads to the attenuation of the up-regulated protein expression of TGase 1 by UVB irradiation. Therefore, to determine whether MSK1 inhibition or inactivation is substantially involved with the attenuated protein expression of TGase 1, as observed for AX, we next examined the effect of the MSK1 inhibitor H89 [59] on the accentuated protein expression of TGase 1 in UVB-exposed human keratinocytes because MSK1 belongs to the PKA family and its activity is inhibited by H89 [51]. Treatment with H89 post-irradiation significantly abrogated the increased protein expression level of TGase 1 as well as COX2 in UVB-exposed HPKs in a dose-dependent manner, which was accompanied by the abrogating effects on the increased phosphorylation of NFκBp65Ser276 and CREBSer133 but not Thr565/Ser376/Ser360 MSK1 (data not shown). Since the expression of COX2 is mediated via the NFκB pathway [21], the H89 inhibition study strongly indicates that the attenuated expression of TGase 1 is mediated through the specific interruption of the p38/MSK1/Ser276NFκBp65 axis. Although the abrogated protein expression of TGase 1 may also be attributable to the inhibition of cAMP-dependent PKA by H89, this possibility can be ruled out by the fact that drugs known to enhance cAMP levels, such as PGE_2_, cholera toxin and dibutyryl cAMP, decrease the production of NFκB pathway-triggered proteins, such as IL-8 and COX2, in irradiated cells by down-regulating NFκB activation in response to UVB radiation [60]. This is an indication that H89 treatment does not abrogate the UVB-stimulated protein expression of TGase 1 as well as COX2 via interruption of the cAMP/PKA pathway. To further verify that association with MSK1 inactivation, we next examined the effect of silencing MSK1 mRNA and found that silencing MSK1 significantly abrogates the UVB-induced accentuation of TGase 1 protein levels.

A similar mechanism of action on MSK1 was seen in human keratinocytes and in dendritic cells by dimethyfumarate (DMF) as an anti-psoriatic agent [61] and a therapeutic agent for multiple sclerosis [62], respectively, as well as in mouse skin by docosahexaenoic acid (DHA) [63]. In human keratinocytes, whereas protein kinases, including p38 MAPK and ERK p44/p42 MAPK, are not influenced by DMF, the IL-1β-induced activation of both MSK1 and MSK2 is specifically attenuated [61]. DMF interrupts the maturation of dendritic cells by down-regulating inflammatory cytokine production (IL-12 and IL-6) where NFκB signaling is decreased by DMF through the suppression of ERK1/2 and its downstream kinase MSK1 [62]. MSK1 suppression results in decreased NFκBp65 phosphorylation at Ser276 and reduced histone phosphorylation at Ser10. DMF also impairs NFκB signaling via reduced levels of NFκBp65 nuclear translocation by acting as an alternative mechanism, probably including ERK inhibition which is distinct from that caused by the post-irradiation treatment with AX. DMF [64] or MSK1 silencing [65] has also been shown to inhibit NFκB-mediated expression of IL-8 in LPS-stimulated peripheral blood mononuclear cells or in human keratinocytes, respectively. Available evidence indicates that, after dietary supplementation with DMF, a significant modulation of intracellular levels of detoxifying enzyme systems, such as the reduced glutathione (GSH)-oxidized glutathione-disulfide (GSSG) system is provoked to up-regulate the GSH level [66]. Since NFκB activation has been shown to be efficiently down-regulated by increased GSH levels [67], it is likely that, as seen for the antioxidants AX and DHA, DMF has a distinct influence on the intracellular redox balance to affect the phosphorylation of MSK1 through increased levels of GSH. This mode of action may resemble the inhibitory effect of AX on the increased phosphorylation of MSK1 in UVB-exposed human keratinocytes.

**Fig 14:**
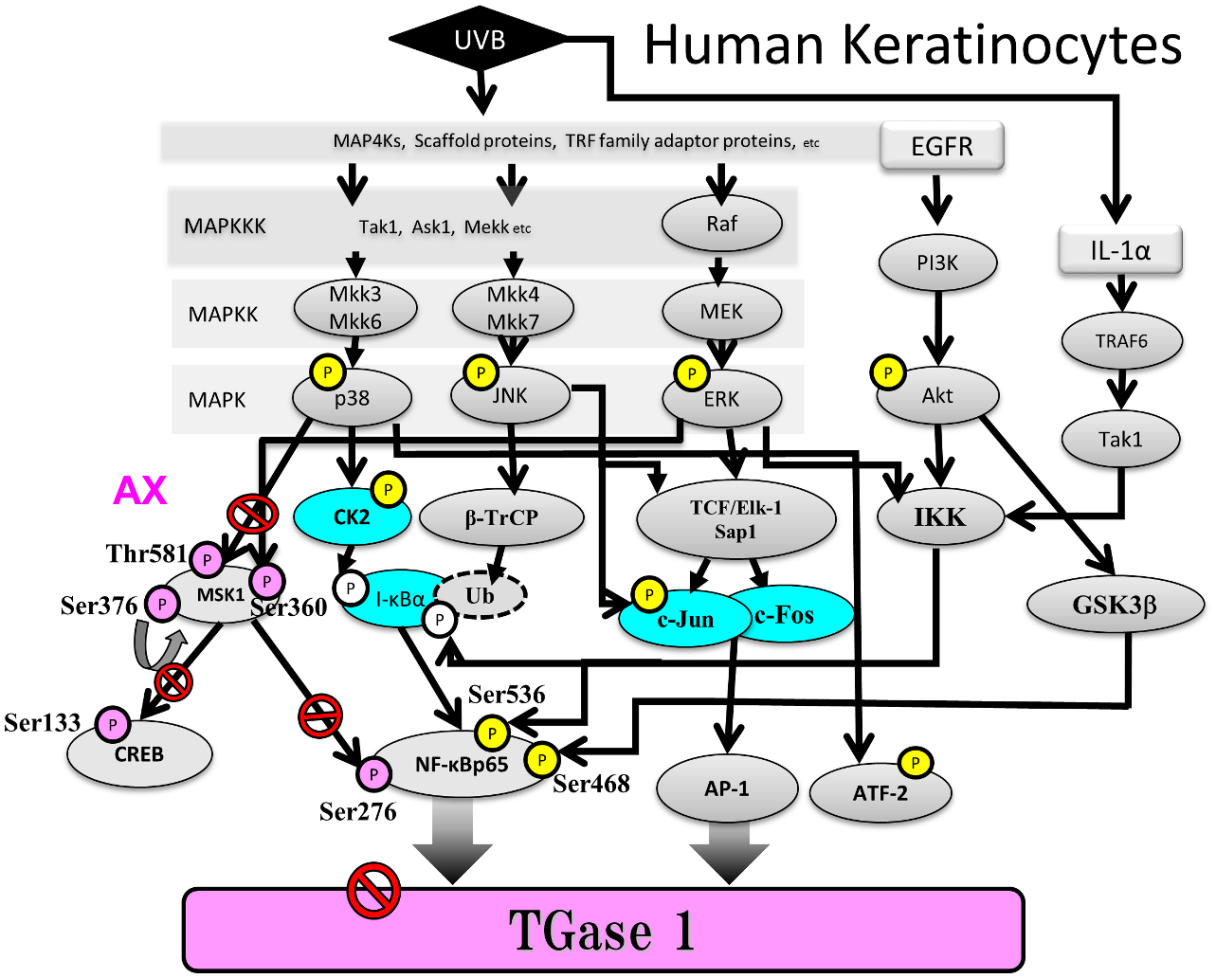
Intracellular signaling pathways leading to the UVB-induced increase in the expression of TGase 1 and the site of inhibition by the post-irradiation treatment of human keratinocytes with AX. The encircled P’s colored yellow and pink represent UVB-increased and non- or significantly abrogated phosphorylations, respectively, in each signaling molecule by the post-irradiation treatment with AX. The blue-colored signaling molecules show an increase or a decrease in each protein level by the post-irradiation treatment with AX in UVB-exposed human keratinocytes.

In conclusion, to our knowledge, this is the first detailed in vitro study of the signaling mechanisms involved in TGase 1 expression during epidermal differentiation subsequent to UVB exposure. Since MSK1 activation is essentially involved in the NFκB signaling pathway, which serves as a nuclear kinase for Ser276 NFκBp65 after its translocation into the nucleus [51], the sum of our findings suggests that, as depicted in Fig 14, the UVB-stimulated expression of TGase 1 is mediated predominantly via the NFκB signaling pathway, which is evidenced by their significant attenuation by MSK1 silencing, H89 and AX through specific interruption of the p38/MSK1/NFκBp65 Ser276 axis.
